# Association between oxidative balance score and gallstone in US adults: a cross-sectional study

**DOI:** 10.3389/fragi.2025.1621107

**Published:** 2025-07-15

**Authors:** Xiaoya Chen, Xiongwei Huo, Changchun Ye, Zhengshui Xu, Zilu Chen, Shiyuan Liu

**Affiliations:** ^1^ Department of Thoracic Surgery, The Second Affiliated Hospital of Xi’an Jiaotong University, Xi’an, China; ^2^ Department of General Surgery, The First Affiliated Hospital of Xi’an Jiaotong University, Xi’an, China

**Keywords:** oxidative balance score, gallstone, NHANES, dietary, lifestyle

## Abstract

**Introduction:**

The Oxidative Balance Score (OBS) serves as a means to evaluate the systemic oxidative stress status, where the higher OBS score indicates a greater exposure to antioxidants. Few studies have delved into the connection between the systemic oxidative stress status and gallstone.

**Methods:**

A total of 4,376 from the NHANES participants were included in this cross-sectional analysis using 2017–2020 survey cycles. Gallstone was diagnosed by the Patient Health Questionnaire. OBS was scored by 16 dietary factors and 4 lifestyle factors. Logistic regression, subgroup analysis and restricted cubic splines (RCS) were used to assess the association between OBS and gallstone.

**Results:**

In a sample comprising 4,376 individuals, logistic regression illuminated a negative association between OBS and gallstone [OR = 0.96 (0.94, 0.98), p < 0.001]. Compared to the lowest quartile of OBS, the fully adjusted ORs for the highest quartile of total OBS and gallstone were 0.65 (0.45, 0.95), p=0.025. Robust associations were also discerned between gallstone and both dietary and lifestyle OBS. The results of the subgroup analysis showed significant differences in the association between lifestyle OBS and gallstone with respect to age and marital status. RCS analysis indicated a significant linear relationship between OBS and gallstone.

**Discussion:**

Our study exhibited a reverse relationship between OBS and the prevalence of gallstone among American adults, which provided a theoretical foundation for designing personalized dietary regimens and lifestyle modifications to mitigate gallstone formation.

## 1 Introduction

Gallstones are one of the most common and costly of all the gastrointestinal diseases (Marschall and Einarsson, 2007) and >20% of people with gallstones will develop symptoms in their lifetime (including biliary colic or infections), usually in adulthood (Lammert et al., 2016). Laparoscopic cholecystectomy is considered the most cost-effective management strategy in the treatment of symptomatic gallstones (Bellows et al., 2005). They give rise to substantial health and economic burden. The pathogenesis of gallstones arises from multiple contributors, encompassing dynamic interactions among gender-related predispositions, heritable factor, modifiable behavioral patterns, and disease-mediated metabolic alterations (Shabanzadeh, 2018). Oxidative stress is regarded as one of the primary risk factors contributing to gallstone formation (Koppisetti et al., 2008). Therefore, investigating the role of oxidative stress in the formation and progression of gallstones is crucial for understanding the underlying pathophysiological mechanisms and developing effective preventive strategies.

Oxidative stress is defined as an imbalance between production of free radicals and reactive metabolites, so-called oxidants or reactive oxygen species (ROS), and their elimination by protective mechanisms, referred to as antioxidants (Reuter et al., 2010). This imbalance cause damage to cellular macromolecules such as DNA, lipids and proteins, eventually leading to necrosis and apoptotic cell death (Senoner and Dichtl, 2019). The process can be influenced by various modifiable factors, such as diet, smoking, or medicines (Poljsak et al., 2013). So, the level of oxidative stress in the body is complex. We believe that exposure to a single factor or solely dietary factors may not fully reflect the body’s role in maintaining overall oxidative balance, and a comprehensive evaluation of multiple factor combinations may be more meaningful. OBS has a significant advantage in combining various oxidants and antioxidants in diet and lifestyle, and may be a more accurate overall indicator of oxidative stress (Hernández-Ruiz et al., 2022).

There has been a series of literature to explore the relationship between OBS and depression (Liu et al., 2023), kidney stone (Ke et al., 2023), diabetes (Wu et al., 2023) and other diseases (Xu et al., 2024; Qu, 2023; Hu et al., 2024), but there is no article to discuss the link between gallstones and OBS. Therefore, this study aimed to assess the association between OBS and gallstone among adults in the United States, using NHANES data, for aiding the development of new preventive behavior for the diseases.

## 2 Materials and methods

### 2.1 Study population

This study was a cross-sectional study and the data were primarily obtained from NHANES, a program that is widely used by researchers to assess the health and nutrition status of adults and children in the United States (Xu et al., 2024). Each participant completed questionnaires and examinations at the Mobile Examination Centers (MECs) and written informed consent was obtained from participants.

**FIGURE 1:**
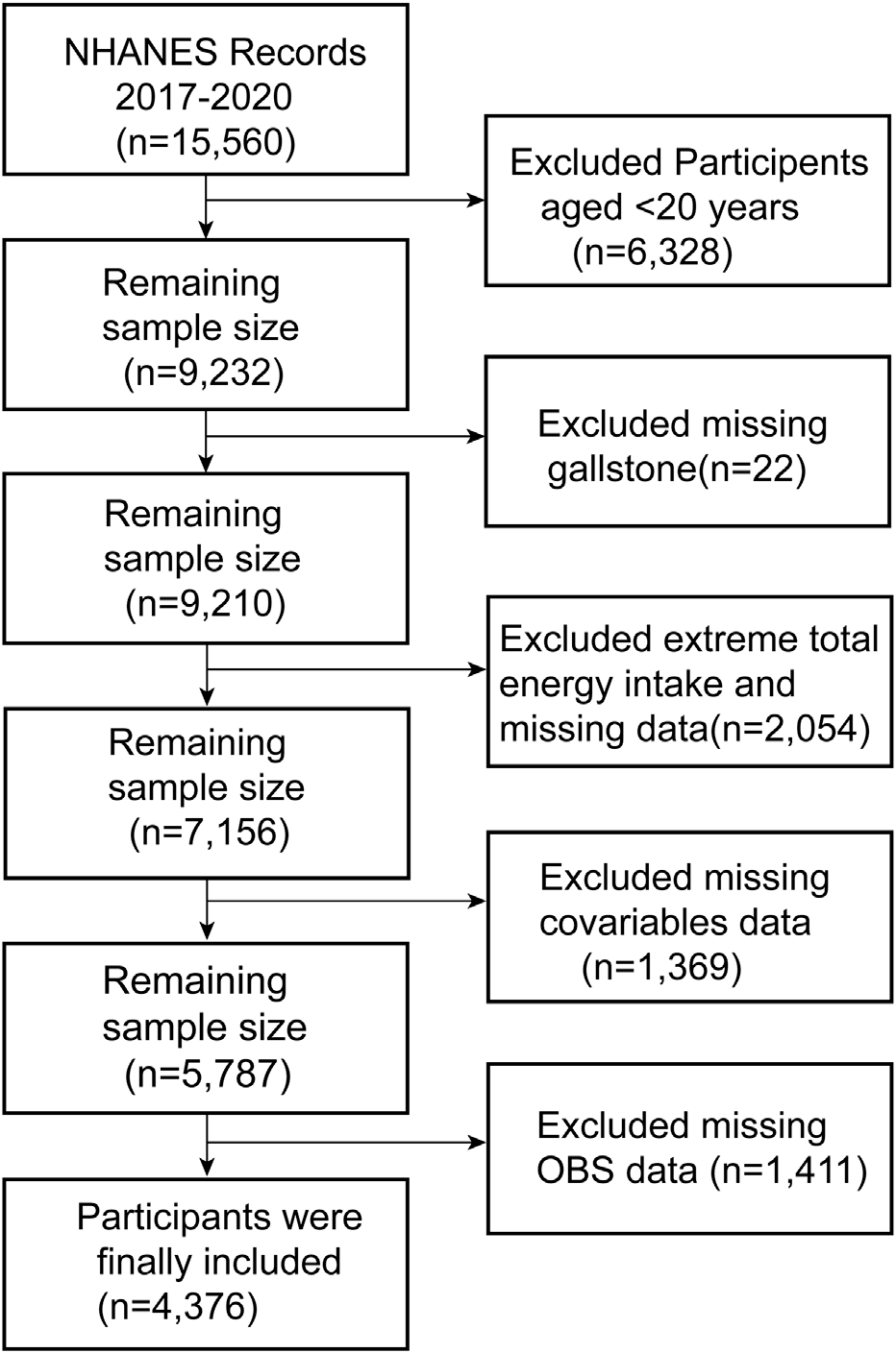
Flowchart showing the selection of the studied population.

The year 2017–2020,which included a total of 15,560 participants was used for the study data because the questionnaire for gallstones was only available during that time period. Since the questionnaire was only administered to adults aged 20 years and older, we removed participants under the age of 20 years, and based on the purpose of our study, we screened the study population, with detailed inclusion and exclusion criteria provided in Figure 1. To be specific, participants with missing gallstone index data (*n* = 22), extreme energy intake (total energy intakes below 800 or above 4,200 kcal/day for males and below 500 or above 3,500 kcal/day for females (Hou et al., 2022)) (*n* = 2,054), missing covariates (*n* = 1,369) and incomplete OBS data (*n* = 1,411) were omitted. As a consequence, only 4,376 participants were eligible for complete case analysis.

### 2.2 Outcome variable

Gallstone cases were identified using the inquiry “Has DR ever said you have gallstones”. Participants who responded affirmatively were classified as having gallstones.

### 2.3 Exposure variable

OBS was determined by integrating 16 dietary factors and 4 lifestyle components, comprising 5 pro-oxidants and 15 antioxidants (Hernández-Ruiz et al., 2019). Dietary OBS, including dietary fiber, total fat, total folate, vitamins (B6, B12, C and E), niacin, carotene, riboflavin, calcium, iron, selenium, copper, magnesium, and zinc, which were collected from the first dietary recalls.

The lifestyle OBS is composed of four components: smoking, alcohol consumption, physical activity, and body mass index (BMI). Serum cotinine levels were used to quantify the smoking factor, which reflects both direct smoking and exposure to second-hand smoking (Tian et al., 2022). Alcohol consumption was categorized into three groups based on different gender: heavy drinkers (≥15 g/d for women and ≥ 30 g/d for men), non-heavy drinkers (0–15 g/d for women and 0–30 g/d for men), and non-drinkers, who were assigned 0, 1, and 2 points, respectively (Zhang et al., 2022). Total physical activity was quantified as the metabolic equivalent of task (MET), calculated based on the accumulated time of transportation and moderate and vigorous activities per week. MET scores = weekly frequency of each physical activity * duration of each physical activity* each physical activity suggested MET Scores (Tian et al., 2022). Except for alcohol consumption, the other OBS components were categorized by gender and divided into tertiles. Scores were assigned to antioxidants in tertiles 1 to 3 as 0, 1, and 2, respectively, while scores were assigned to pro-oxidants in the opposite order (Supplementary Table S1). A higher OBS score indicates a more substantial antioxidant effect. In cases where OBS components were missing, the corresponding component was assigned a score of 0, regardless of its antioxidant or pro-oxidant nature.

### 2.4 Covariates

Potential covariates that could confound the association between OBS and gallstones were summarized in the multivariable-adjusted model. Covariates in our study included sex (male, female), age (<40, 40–60, ≥60), ethnicity (Hispanic, non-Hispanic white, non-Hispanic black and other races), education level (below high school, high school, above high school), poverty income ratio (PIR, <1.3, 1.3–3.5, or ≥ 3.5), marital status (married or living with a partner, widowed/divorced/separated, never married), hypertension, diabetes, asthma, coronary heart disease (CHD). The definition of the comorbidities was collected in Supplementary Table S2. In addition, we incorporated total cholesterol (TC; mg/dL) and total energy intake (kcal). More details on variable collection methods can be found in the NHANES Survey Methods and Analysis Guide.

### 2.5 Statistical analyses

Statistical analyses were conducted utilizing appropriate NHANES sampling weights according to NHANES recommendations and guidelines, accounting for the complicated multistage entire cohort survey. Continuous variables were expressed as mean ± standard error, while categorical variables were displayed as unweighted counts and weighted proportions. ANOVA tests were used for comparing continuous variables, and chi-square tests were employed to examine statistical differences in categorical variables between groups. Then, we investigated the association between OBS and gallstones in 3 different models using multivariate logistic regression models: model 1 was a crude model without adjusting covariates, model 2 was adjusted for age, gender, and race, and model 3 was adjusted for all variables. Identical analyses were conducted for dietary OBS and lifestyle OBS with gallstone. We further assessed the heterogeneity between OBS and gallstones by subgroup analysis, including the following variables: age, gender, race, marital status, PIR, education level, diabetes, hypertension, asthma and CHD. Interaction tests were used to examine the consistency of the relationships between the different subgroups. Sensitivity analysis was performed by systematically eliminating each factor from the adjusted model 3. Finally, a restricted cubic spline (RCS) curve was performed to investigate the potentially nonlinear association between exposure and outcome.

Statistical analyses were two-sided, and *p* < 0.05 was considered statistically significant. All analyses were performed utilizing R (version 4.2.0) and EmpowerStats software (http://www.empowerstats.com).

## 3 Results

### 3.1 Baseline characteristics

A total of 4,376 subjects with complete information were included in the study after combining covariates. Table 1 presents the baseline characteristics of the participants, stratified by quartiles of total OBS. Among them, 428 had gallstones, accounting for a prevalence rate of 10.29%. The mean age of the subjects was 47.34 ± 0.67 years, with 49.78% being female and the majority being non-Hispanic whites (67.62%). In the higher quartile, we also observed an increase in the consumption of energy, along with higher levels of education, wealth among individuals and those who married or living with partner (all *p* < 0.001). However, the differences in gender, asthma, CHD and diabetes were not statistically significant. Supplementary Table S3 presents additional baseline characteristic according to the absence or presence of gallstone. Individuals with gallstone tended to be older, predominantly female and had lower OBS scores. The gallstone group also have a higher BMI and exhibited a more significant burden of comorbidities. The educational level showed no significant difference between the two groups.

**TABLE 1:** Characteristic of study participants by OBS quartile (N = 4,376).

Characteristic	Overall	Q1 [0.14)	Q2 [14.20)	Q3 [20.26)	Q4≥ 26	*p-*value
	N = 4,376	N = 1,078	N = 939	N = 1,223	N = 1,136	
Age (years), Mean ± SE	47.34 ± 0.67	45.40 ± 1.09	47.00 ± 1.03	48.24 ± 0.73	48.07 ± 1.00	0.058
Gender, n (%)						0.323
Male	2,201 (50.22)	520 (47.16)	488 (49.57)	622 (49.89)	571 (53.25)	
Female	2,175 (49.78)	558 (52.84)	451 (50.43)	601 (50.11)	565 (46.76)	
Race, n (%)						**<0.001**
Mexican American	500 (7.73)	78 (5.57)	95 (6.58)	152 (7.76)	175 (10.10)	
Non-Hispanic White	1,698 (67.62)	367 (63.44)	384 (68.58)	493 (70.19)	454 (67.28)	
Non-Hispanic Black	1,045 (9.39)	375 (15.41)	236 (10.16)	256 (7.90)	178 (6.02)	
Other	1,133 (15.26)	258 (15.59)	224 (14.68)	322 (14.15)	329 (16.61)	
Education level, n (%)						**<0.001**
Below high school	628 (7.87)	167 (9.53)	141 (7.36)	170 (7.90)	150 (7.02)	
High school	1,011 (25.83)	321 (35.61)	225 (26.30)	262 (22.92)	203 (21.43)	
High school above	2,737 (66.30)	590 (54.86)	573 (66.33)	791 (69.18)	783 (71.55)	
PIR group, n (%)						**<0.001**
≤1.3	1,129 (16.34)	367 (23.56)	237 (16.65)	286 (14.15)	239 (13.16)	
1.3–3.5	1,646 (33.22)	422 (36.52)	374 (35.28)	464 (32.25)	386 (30.33)	
>3.5	1,601 (50.44)	289 (39.92)	328 (48.07)	473 (53.60)	511 (56.52)	
Marital status, n (%)						**<0.001**
Married/Living with Partner	2,630 (64.34)	540 (53.43)	575 (65.68)	776 (67.96)	739 (67.51)	
Widowed/Divorced/Separated	891 (16.53)	264 (19.27)	195 (17.13)	229 (14.76)	203 (15.95)	
Never married	855 (19.12)	274 (27.29)	169 (17.19)	218 (17.28)	194 (16.54)	
Energy intake (kcal), Mean ± SE	2,107.23 ± 12.92	1,452.12 ± 23.53	1871.68 ± 27.67	2,211.33 ± 31.98	2,645.18 ± 25.72	**<0.001**
Cholesterol (mmol/L),Mean ± SE	4.86 ± 0.04	4.75 ± 0.07	5.02 ± 0.05	4.90 ± 0.05	4.79 ± 0.04	**<0.001**
Cotinine (ng/mL),Mean ± SE	50.14 ± 5.00	88.86 ± 14.56	56.17 ± 6.47	37.48 ± 4.71	30.83 ± 3.37	**<0.001**
Alcohol (g/d), Mean ± SE	1.99 ± 0.07	2.05 ± 0.11	2.03 ± 0.12	1.90 ± 0.10	2.02 ± 0.12	0.762
MET (minute/week), Mean ± SE	4,175.42 ± 172.66	4,280.64 ± 238.72	4,354.44 ± 404.37	3,856.30 ± 215.56	4,297.16 ± 354.56	0.116
BMI(kg/m^2^),Mean ± SE	29.46 ± 0.19	30.42 ± 0.40	29.85 ± 0.37	29.77 ± 0.40	28.17 ± 0.28	**<0.001**
Dietary OBS, Mean ± SE	16.70 ± 0.16	7.36 ± 0.10	13.02 ± 0.09	18.48 ± 0.07	24.31 ± 0.08	**<0.001**
Lifestyle OBS, Mean ± SE	4.13 ± 0.03	3.84 ± 0.05	3.96 ± 0.06	4.06 ± 0.06	4.55 ± 0.05	**<0.001**
Asthma, n (%)						0.099
Yes	684 (14.13)	186 (17.12)	154 (14.99)	184 (13.25)	160 (12.25)	
No	3,692 (85.87)	892 (82.88)	785 (85.01)	1,039 (86.75)	976 (87.75)	
CHD, n (%)						0.820
Yes	176 (3.86)	42 (4.15)	42 (3.15)	53 (3.78)	39 (4.24)	
No	4,200 (96.14)	1,036 (95.85)	897 (96.85)	1,170 (96.22)	1,097 (95.76)	
Hypertension, n (%)						**0.038**
Yes	1,534 (29.66)	424 (31.91)	338 (31.59)	432 (30.76)	340 (25.50)	
No	2,842 (70.34)	654 (68.09)	601 (68.41)	791 (69.24)	796 (74.50)	
Diabetes, n (%)						0.099
Yes	785 (13.87)	225 (16.95)	175 (15.13)	217 (12.59)	168 (12.06)	
No	3,591 (86.13)	853 (83.05)	764 (84.87)	1,006 (87.41)	968 (87.94)	
Gallstone, n (%)						0.334
Yes	428 (10.29)	959 (87.81)	826 (88.94)	1,115 (89.83)	1,048 (91.53)	
No	3,948 (89.71)	119 (12.19)	113 (11.06)	108 (10.17)	88 (8.47)	

Q1: 0 ≤ Total OBS<14; Q2: 14 ≤ Total OBS<20; Q3: 20 ≤ Total OBS<26; Q4 Total OBS ≥ 26.

For continuous variables: survey-weighted mean (SE), P-value was by survey-weighted linear regression.

For categorical variables: survey-weighted percentage (95% CI), P-value was by survey-weighted Chi-square test.

SE, standard error; PIR, income to poverty ratio; CHD, coronary heart disease; BMI, body mass index; MET, metabolic equivalent; OBS, oxidative balance score.

### 3.2 Relationship between total OBS and gallstone

To explore the relationship between total OBS and gallstone, we performed the logistic regression analyses. As shown in Table 2, when OBS was treated as a continuous variable, we observed that it was significantly associated with a lower incidence of developing gallstone in both crude model (OR = 0.97 (0.96, 0.99), *p* < 0.001) and fully adjusted model (OR = 0.96 (0.94, 0.98), *p* < 0.001). When considering OBS as a categorical variable, in the fully adjusted Model 3, the highest quartile of OBS was more negatively associated with the risk of gallstone than the lowest quartile of OBS (OR = 0.65 (0.45, 0.95), *p* = 0.025), and maintained relative stability across models. When compared to the reference category of the first OBS quartile, the OR (95% CI) for the third OBS quartile was 0.72 (0.52.0.99), *p* = 0.045. The observed trend was highly significant (*p* = 0.006). To sum up, this study revealed a significant inverse correlation between total OBS and gallstone, with individuals in the highest OBS quartile exhibiting a 35% lower risk compared to those in the lowest quartile. In sensitivity analyses, excluding any of the OBS components had no significant effect on the gallstone, indicating the robustness of our finding (Supplementary Table S4).

**TABLE 2:** Association between the OBS and gallstone prevalence based on logistic regression analysis.

Exposures	Model 1		Model 2		Model 3	
	OR [95%CI]	*p*	OR [95%CI]	*p*	OR [95%CI]	*p*
Total OBS	0.97 (0.96, 0.99)	<0.001	0.97 (0.95, 0.98)	<0.001	0.96 (0.94, 0.98)	<0.001
Total OBS quantile						
Q1	ref		ref		ref	
Q2	1.10 (0.84, 1.45)	0.485	1.06 (0.80, 1.41)	0.694	1.08 (0.80, 1.45)	0.607
Q3	0.78 (0.59, 1.03)	0.077	0.70 (0.53, 0.94)	0.016	0.72 (0.52, 0.99)	0.045
Q4	0.68 (0.51, 0.90)	0.008	0.62 (0.46, 0.83)	0.002	0.65 (0.45, 0.95)	0.025
P for trend		0.001		<0.001		0.006
Dietary OBS	0.98 (0.96, 0.99)	0.002	0.97 (0.96, 0.99)	<0.001	0.97 (0.95, 0.99)	0.006
Dietary OBS quantile						
Q1	ref		ref		ref	
Q2	1.11 (0.84, 1.46)	0.456	1.03 (0.78, 1.37)	0.83	1.03 (0.77, 1.39)	0.824
Q3	0.79 (0.60, 1.04)	0.092	0.71 (0.53, 0.94)	0.018	0.72 (0.52, 1.00)	0.048
Q4	0.72 (0.54, 0.96)	0.027	0.65 (0.48, 0.87)	0.004	0.67 (0.46, 0.99)	0.044
P for trend		0.005		<0.001		0.013
Lifestyle OBS	0.85 (0.79, 0.92)	<0.001	0.84 (0.77, 0.91)	<0.001	0.86 (0.79, 0.93)	<0.001
Lifestyle OBS quantile						
Q1	ref		ref		ref	
Q2	1.03 (0.74, 1.42)	0.883	1.04 (0.74, 1.46)	0.823	1.09 (0.77, 1.53)	0.631
Q3	0.81 (0.58, 1.11)	0.191	0.74 (0.53, 1.04)	0.079	0.79 (0.56, 1.11)	0.166
Q4	0.62 (0.45, 0.85)	0.003	0.59 (0.43, 0.83)	0.002	0.65 (0.47, 0.92)	0.013
P for trend		<0.001		<0.001		<0.001

OR, odds ratio; CI, confidence intervals; OBS, oxidative balance score.

The model 1 was the crude model.

The model 2 was adjusted by gender, age, race.

The model 3 was adjusted by gender; age; race; marriage; education, energy; PIR; cholesterol; asthma; CHD; hypertension; diabetes.

### 3.3 Association of dietary OBS and lifestyle OBS with gallstone

To investigate the potential differential contributions of lifestyle OBS and dietary OBS to gallstone pathogenesis, we conducted logistic regression analyses parallel to the total OBS evaluation, as presented in Table 2. Both dietary and lifestyle OBS subtypes exhibited statistically significant negative associations with gallstone. Higher levels of continuous dietary and lifestyle OBS consistently showed correlations with a reduced incidence of gallstone (*p* < 0.05). In the fully adjusted Model 3, the fourth quartile of dietary OBS significantly impacted gallstone (OR = 0.67 (0.46, 0.99), *p* = 0.044). Similarly, lifestyle OBS also demonstrated a persistent effect, with the fourth quartile indicating a negative correlation with gallstone (OR = 0.65 (0.47, 0.92), *p* = 0.013). The trend test indicated that the downward trend was statistically significant (*p* for trend <0.001).

### 3.4 Subgroup analysis

Further, to understand the association between total OBS and gallstone in different populations, we performed the subgroup analyses by fully adjusted multivariate logistic regression analyses stratified by age, race, gender, education levels, PIR, and comorbidities including asthma, CHD, diabetes and hypertension. As showed in Table3, the interaction term was no evidence of effect modification. A significant inverse association was observed in younger age groups (*p* = 0.022), other race (*p* = 0.004) and individuals with education below high school (*p* = 0.070). While point estimates suggested a stronger association in females (OR = 0.96) versus males (OR = 0.97), the interaction term for gender was non-significant (p for interaction = 0.650), indicating no statistical evidence of differential effects by sex.

**TABLE 3:** Subgroup analysis of the association between total oxidative balance score and gallstone.

Subgroup	N (%)	OR (95%CI)	*p value*	*p* for interaction
Age (year)				0.338
≤40	1,529 (34.94)	0.95 (0.91–0.99)	0.022	
40–60	1,531 (34.99)	0.96 (0.92–0.98)	0.010	
>60	1,316 (30.07)	0.99 (0.96–1.02)	0.471	
Gender				0.650
male	2,201 (50.30)	0.97 (0.93–1.00)	0.086	
female	2,175 (49.70)	0.96 (0.94–0.99)	0.002	
Race				0.516
Mexican American	500 (11.43)	0.97 (0.91–1.04)	0.408	
Non-Hispanic White	1,698 (38.80)	0.98 (0.95–1.01)	0.245	
Non-Hispanic Black	1,045 (23.88)	0.97 (0.92–1.01)	0.149	
Other	1,133 (25.89)	0.94 (0.91–0.98)	0.004	
Education level, n (%)				0.415
Below high school	628 (14.35)	0.95 (0.90–1.00)	0.070	
High school	1,011 (23.10)	1.00 (0.96–1.04)	0.983	
High school above	2,737 (62.55)	0.96 (0.93–0.98)	<0.001	
PIR group, n (%)				0.675
≤1.3	1,129 (25.80)	0.96 (0.92–1.00)	0.056	
1.3–3.5	1,646 (37.61)	0.98 (0.94–1.01)	0.129	
>3.5	1,601 (36.59)	0.96 (0.93–0.99)	0.014	
Marital status, n (%)				0.055
Married/Living with Partner	2,630 (60.10)	0.97 (0.94–0.99)	0.011	
Widowed/Divorced/Separated	891 (20.36)	0.96 (0.92–1.00)	0.069	
Never married	855 (19.54)	0.96 (0.91–1.02)	0.165	
Asthma				0.611
Yes	684 (15.63)	0.94 (0.90–0.99)	0.025	
No	3,692 (84.37)	0.97 (0.95–0.99)	0.005	
CHD				0.114
Yes	176 (4.02)	0.92 (0.85–1.01)	0.067	
No	4,200 (95.98)	0.97 (0.95–0.99)	0.003	
Hypertension, n (%)				0.665
Yes	1,534 (35.05)	0.97 (0.94–1.00)	0.044	
No	2,842 (64.95)	0.96 (0.94–0.99)	0.008	
Diabetes, n (%)				0.147
Yes	785 (17.94)	0.97 (0.93–1.02)	0.221	
No	3,591 (82.06)	0.96 (0.94–0.99)	0.001	

Gender; age; race; marriage; education, energy; PIR; cholesterol; asthma; CHD; hypertension and diabetes were adjusted.

To further explore potential effect modifiers, we conducted subgroup analyses between lifestyle OBS (Table 4) or dietary OBS (Table 5) and gallstone. A significant interaction by age (*p* for interaction<0.001) and marital status (*p* for interaction = 0.013) were observed in the relationship between lifestyle OBS and gallstone. The protective effect of lifestyle OBS was strongest in younger group (OR = 0.75 (0.62.0.91), *p* = 0.003), which is consistent with total OBS. The inverse association was markedly stronger in individuals with asthma (OR = 0.69 (0.57.0.84), *p* < 0.001) compared to those without asthma (*p* for interaction<0.001). Except age, marital status and asthma, our results showed that *p*-value for interaction was >0.05 among other subgroups, indicating consistency in our findings.

**TABLE 4:** Subgroup analysis of the association between lifestyle oxidative balance score and gallstone.

Subgroup	N (%)	OR (95%CI)	*p value*	*p* for interaction
Age (year)				<0.001
≤40	1,529 (34.94)	0.75 (0.62–0.91)	0.003	
40–60	1,531 (34.99)	0.80 (0.69–0.91)	0.001	
>60	1,316 (30.07)	0.99 (0.88–1.12)	0.869	
Gender				0.856
male	2,201 (50.30)	0.85 (0.74–0.98)	0.023	
female	2,175 (49.70)	0.86 (0.78–0.95)	0.003	
Race				0.202
Mexican American	500 (11.43)	0.78 (0.61–1.00)	0.047	
Non-Hispanic White	1,698 (38.80)	0.92 (0.82–1.04)	0.195	
Non-Hispanic Black	1,045 (23.88)	0.82 (0.67–1.00)	0.048	
Other	1,133 (25.89)	0.86 (0.73–1.01)	0.069	
Education level, n (%)				0.207
Below high school	628 (14.35)	0.81 (0.64–1.01)	0.061	
High school	1,011 (23.10)	0.90 (0.77–1.06)	0.219	
High school above	2,737 (62.55)	0.86 (0.78–0.95)	0.004	
PIR group, n (%)				0.707
≤1.3	1,129 (25.80)	0.91 (0.77–1.07)	0.249	
1.3–3.5	1,646 (37.61)	0.87 (0.76–0.99)	0.031	
>3.5	1,601 (36.59)	0.84 (0.73–0.96)	0.012	
Marital status, n (%)				0.013
Married/Living with Partner	2,630 (60.10)	0.89 (0.80–0.98)	0.022	
Widowed/Divorced/Separated	891 (20.36)	0.90 (0.77–1.05)	0.178	
Never married	855 (19.54)	0.71 (0.56–0.90)	0.005	
Asthma				<0.001
Yes	684 (15.63)	0.69 (0.57–0.84)	<0.001	
No	3,692 (84.37)	0.91 (0.84–1.00)	0.051	
CHD				0.623
Yes	176 (4.02)	0.87 (0.63–1.19)	0.372	
No	4,200 (95.98)	0.87 (0.80–0.94)	<0.001	
Hypertension, n (%)				0.163
Yes	1,534 (35.05)	0.85 (0.75–0.95)	0.006	
No	2,842 (64.95)	0.89 (0.79–0.99)	0.033	
Diabetes, n (%)				0.077
Yes	785 (17.94)	0.87 (0.74–1.03)	0.101	
No	3,591 (82.06)	0.87 (0.79–0.95)	0.003	

Gender; age; race; marriage; education, energy; PIR; cholesterol; asthma; CHD; hypertension and diabetes were adjusted.

**TABLE 5:** Subgroup analysis of the association between dietary oxidative balance score and gallstone.

Subgroup	N (%)	OR (95%CI)	*p* value	*p* for interaction
Age (year)				0.624
≤40	1,529 (34.94)	0.96 (0.92–1.01)	0.096	
40–60	1,531 (34.99)	0.97 (0.93–1.00)	0.064	
>60	1,316 (30.07)	0.99 (0.96–1.02)	0.477	
Gender				0.653
male	2,201 (50.30)	0.98 (0.94–1.02)	0.231	
female	2,175 (49.70)	0.97 (0.95–0.99)	0.016	
Race				0.628
Mexican American	500 (11.43)	0.99 (0.92–1.06)	0.769	
Non-Hispanic White	1,698 (38.80)	0.99 (0.96–1.02)	0.380	
Non-Hispanic Black	1,045 (23.88)	0.97 (0.93–1.02)	0.313	
Other	1,133 (25.89)	0.95 (0.91–0.99)	0.009	
Education level, n (%)				0.564
Below high school	628 (14.35)	0.96 (0.90–1.02)	0.152	
High school	1,011 (23.10)	1.01 (0.96–1.05)	0.749	
High school above	2,737 (62.55)	0.96 (0.94–0.99)	0.004	
PIR group, n (%)				0.667
≤1.3	1,129 (25.80)	0.96 (0.92–1.01)	0.086	
1.3–3.5	1,646 (37.61)	0.98 (0.95–1.02)	0.302	
>3.5	1,601 (36.59)	0.97 (0.93–1.00)	0.052	
Marital status, n (%)				0.036
Married/Living with Partner	2,630 (60.10)	0.97 (0.95–1.00)	0.037	
Widowed/Divorced/Separated	891 (20.36)	0.97 (0.93–1.01)	0.127	
Never married	855 (19.54)	0.98 (0.92–1.04)	0.461	
Asthma				0.214
Yes	684 (15.63)	0.97 (0.92–1.02)	0.179	
No	3,692 (84.37)	0.97 (0.95–0.99)	0.015	
CHD				0.189
Yes	176 (4.02)	0.93 (0.85–1.01)	0.091	
No	4,200 (95.98)	0.98 (0.95–1.00)	0.025	
Hypertension, n (%)				0.975
Yes	1,534 (35.05)	0.98 (0.95–1.01)	0.160	
No	2,842 (64.95)	0.97 (0.94–1.00)	0.024	
Diabetes, n (%)				0.197
Yes	785 (17.94)	0.98 (0.94–1.03)	0.393	
No	3,591 (82.06)	0.97 (0.95–0.99)	0.010	

Gender; age; race; marriage; education, energy; PIR; cholesterol; asthma; CHD; hypertension and diabetes were adjusted.

### 3.5 RCS analysis

**FIGURE 2:**
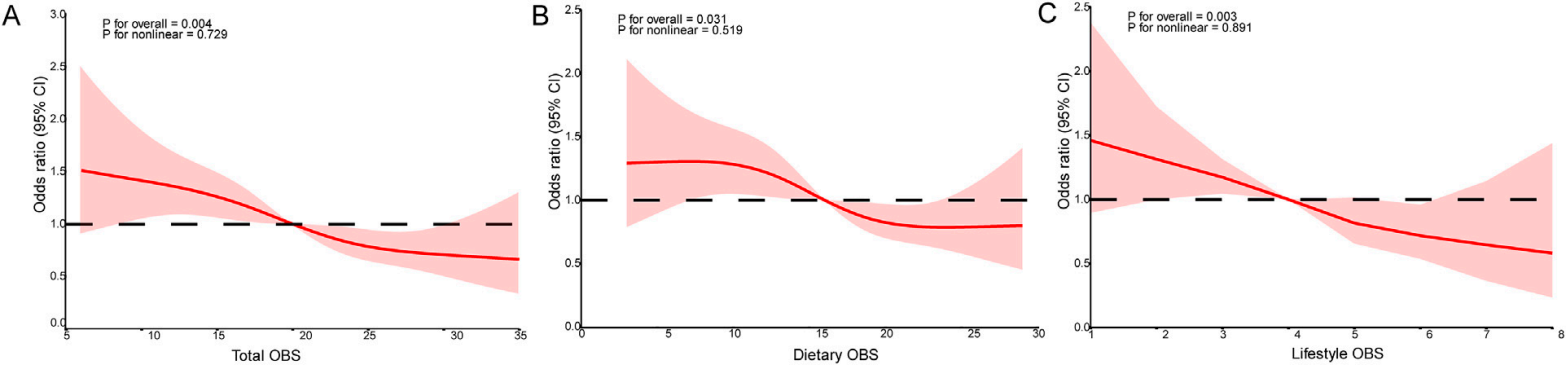
RCS analysis revealed the potential non-linear relationship between OBS and gallstones and it was adjusted by gender, age, race, marriage, education, energy, PIR, cholesterol, asthma, CHD, hypertension and diabetes. **(A)** The RCS curves of the association between total OBS and gallstone. **(B)** The RCS curves of the association between dietary OBS and gallstone. **(C)** The RCS curves of the association between lifestyle OBS and gallstone. RCS, restricted cubic spline, OBS, oxidative balance score; OR, odds ratio; 95% CI, 95% confidence interval; PIR, income to poverty ratio; CHD, coronary heart disease.

To investigate potential nonlinear associations of gallstone risk with overall OBS and its two subcomponents (dietary OBS and lifestyle OBS), RCS analyses were implemented adjusting for all relevant covariates. As showed in Figure 2, the overall association between total OBS (Figure 2A) and gallstone risk (*p* for overall = 0.004, *p* for nonlinear = 0.729) was statistically significant. Interestingly, both diet OBS (Figure 2B) and lifestyle OBS (Figure 2C) exhibit the same trend. However, the non-linear component did not improve the model fit, suggesting a linear dose-response relationship.

## 4 Discussion

To investigate the relationship between OBS and gallstones, we analyzed data from the NHANES 2017–2020 comprising 4,376 participants, which is a nationally representative survey population in the United States. Our findings revealed a robust negative association between OBS and gallstones even after adjusting for sociodemographic variables and covariates for comorbidities, which is consistent with recently published studies (Zhu et al., 2025; Xiong et al., 2025). However, our study implemented stricter exclusion criteria compared to prior analyses (e.g., n = 6,196 in reference studies), removing participants with implausible energy intake to mitigate confounding from aberrant dietary reporting. This methodological refinement likely enhances internal validity by excluding metabolically extreme populations whose oxidative balance profiles may non-representatively skew OBS-gallstone associations. Moreover, our subgroup analyses generated hypotheses about potentially stronger effects in younger individuals and females. However, the lack of statistically significant interactions precludes definitive claims about effect modification by these factors. In conclusion, a higher OBS is associated with a lower risk of developing gallstones, which highlights the significance of diet and lifestyle in preventing the onset of gallstones.

Numerous studies have demonstrated that oxidative stress represents a substantial risk factor for a variety of diseases including tumors (Cheng et al., 2016), cerebrovascular diseases (Marlatt et al., 2008), diabetes (Zhang et al., 2020), as well as neurodegenerative diseases like Alzheimer’s disease (Liu et al., 2012). The most common cellular targets for ROS include cell membrane lipids, proteins, and DNA which causes lipid peroxidation, enzymatic dysfunction, and DNA damage, respectively (Fang et al., 2002; Darley-Usmar and Halliwell, 1996; Floyd, 1990). In this study, the inverse association between OBS and gallstone risk aligns with a studies using ESR spectrum method demonstrating that oxidative stress promotes cholesterol supersaturation in bile, a critical step in gallstone pathogenesis (Liu and Hu, 2002). The research by Tranum Kuar and others in North Indian Population has shown that levels of catalase, SOD, and glutathione-related enzymes were decreased in patients with gallstones in comparison to patients without gallstones, which can be attributed to increase oxidative stress (Kaur and Kaur, 2010). Oxidative stress plays a causative role in the bile duct ligation-induced pigment gallstone formation (Shiesh et al., 2000). A study involving North-East Indian population showed that 8-OH-dG, which is an oxidative stress marker, was significantly higher in the gallstone patients at the plasma and DNA level (Singh et al., 2019). The above-mentioned studies all illustrate the inseparable relationship between oxidative stress and gallstones. A study showed that damage to the gallbladder epithelium mediated by free radicals makes the development of both gallbladder inflammation and gallstone formation more likely (Koppisetti et al., 2008). The mechanism of toxicity by oxygen-derived free radicals is via peroxidation of phospholipids; these are major components of the cell membrane which cause cell death due to loss of cell wall integrity (Weiss, 1989). Phospholipids are also major components of the bile required for cholesterol solubilization (Carey and Small, 1978). Peroxidation of biliary phospholipids is, therefore, the likely mechanism of ROS-induced pronucleating activity. There are also studies indicating that bilirubin oxidation is crucial in the formation of gallstones (Sanikidze and Chikvaidze, 2016). Further studies are needed to clarify the mechanisms of gallstone formation.

Our stratified analyses revealed novel modifiers: the inverse association was markedly stronger in individuals with asthma (OR = 0.69 (0.57–0.84), *p* < 0.001, *p* for interaction<0.001). This may imply that oxidative balance modulation exerts amplified protective effects in pro-inflammatory states, warranting mechanistic studies on glutathione pathways in gallstone formation and underscores a potential shared pathophysiology between oxidative stress, chronic inflammation, and gallstone development, as discussed in a review (Reuter et al., 2010). The stronger protective associations of OBS and lifestyle OBS in younger populations (≤60 years) may reflect age-related differences in oxidative stress metabolism. The potential mechanisms may involve age-dependent cellular damage (Genova and Lenaz, 2015; López-Lluch et al., 2015) mediated through dysregulated ROS signaling pathways, compounded by the cumulative impact of multiple comorbidities commonly present in older adult populations. While the overall OBS association did not significantly differ by gender, the numerically stronger effect size in females hypothetically aligns with estrogen’s antioxidant properties (Kim, 2022) that estrogen is a powerful antioxidant, which modulates oxidative stress pathways and gallstone formation through both direct scavenging and stimulating increased expression of antioxidant enzymes (Strehlow et al., 2003). However, non-significant interaction terms (e.g., for gender, education) must be interpreted cautiously. Apparent subgroup differences may reflect type II error due to limited power rather than true biological effect modification. Future studies with larger samples are needed to conclusively examine these associations. A meta-analysis of 10 cohort studies showed an overall 56% increased risk of gallstones among diabetes patients compared with individuals without diabetes (Aune and Vatten, 2016). Several possible mechanisms may explain the association between type 2 diabetes and gallstone disease. For example, hepatic insulin resistance has been shown to directly promote the formation of cholesterol gallstones (Biddinger et al., 2008). Gallbladder hypomotility in diabetes might be an additional predisposing factor for cholesterol gallstone formation (Portincasa et al., 2006).

The robustness of OBS as a composite biomarker derives from its incorporation of both dietary and lifestyle antioxidants with established redox-modulating properties. Fiber may have protective effects against gallstones by reducing the intestinal transit time and reducing the production of bile acids (Marcus and Heaton, 1986). Schwesinger et al. (1999) have shown the protective effect of dietary soluble fiber against cholesterol gallstone formation. Beta-carotene has been shown to suppress lipid peroxidation in mouse models (Iyama et al., 1996). Many vitamins inhibit NO production by iNOS and also directly scavenge ROS and upregulate the activities of antioxidant enzymes (Wu and Meininger, 2002). Vitamin E inhibits ROS-induced generation of lipid peroxyl radicals, thereby protecting cells from peroxidation of PUFA in membrane phospholipids, from oxidative damage of plasma very low-density lipoprotein, cellular proteins, DNA, and from membrane degeneration (Topinka et al., 1989). Magnesium is a cofactor for glucose-6-phosphate dehydrogenase and 6-phosphogluconate dehydrogenase, two pentose-cycle enzymes catalyzing the production of NADPH from NADP^+^. Thus, a deficiency of dietary magnesium reduces glutathione reductase activity and results in radical-induced protein oxidation and marked lesions in tissues (Rock et al., 1995; Stafford et al., 1993). The essential role of selenium in the removal of free radicals and the maintenance of normal human health is epitomized by the etiology of Keshen disease in China (Yang et al., 1983). Lifestyle factors as OBS component also critically modulate gallstone pathogenesis. A case-control study by McMichael et al. demonstrated a significant association between cigarette smoking and gallbladder disease, particularly emphasizing early exposure effects (McMichael et al., 1992). Smoking may promote gallstone pathogenesis through toxic chemical exposure and insulin resistance—a shared pathway linking smoking to both gallstone and type 2 diabetes (Willi et al., 2007), which is a risk factor for gallbladder disease (Shebl et al., 2010). Exercise or physical activity may contribute to ameliorate insulin resistance by improving insulin action and increasing nitric oxide bioavailability as well as by increasing ROS-detoxification and decreasing ROS generation (Ahmadi et al., 2011; Bjork et al., 2012; Belotto et al., 2010). Currently, obesity is a challenging issue worldwide. A study discovered that the expression of antioxidant enzymes is lower in obese individuals (Le Lay et al., 2014). Collectively, the OBS highlights the multifactorial nature of gallstone prevention, emphasizing that combined dietary and lifestyle interventions rather than isolated factors are essential to mitigate the incidence of gallstone.

Clinically, integrating OBS into risk prediction models could enhance early identification of high-risk population such as obese or diabetic individuals particularly. Public health interventions targeting modifiable OBS components for example physical activity and smoking cessation could offer dual benefits for gallstone prevention and cardiometabolic health. Future studies should integrate multi-omics approaches to dissect OBS subcomponents and explore mechanistic links to biliary pathophysiology. However, this study is not without its limitations. Firstly, one shortcoming of the current study is the inability to differentiate between cholesterol gallstones and pigment gallstones, given that these two types have distinct etiologies. However, it's important to note that in the Western world, the overwhelming majority of gallstones are of the cholesterol type. Secondly, the data on diet, physical activity were derived from patient self-reports, potentially introducing recall bias. Thirdly, it is difficult to establish a causal relationship between OBS and gallstone because of the inescapable disadvantage of cross-sectional study. Consequently, it needs more prospectively designed studies to demonstrate the effectiveness of OBS. Fourthly, we fully acknowledge that complete-case analysis may introduce selection bias if data are not missing completely at random. Sensitivity analyses indicate our findings are robust to data variations at some extent. However, we acknowledge that multiple imputation methods might further enhance precision and will be prioritized in future studies.

## 5 Conclusion

In summary, results from NHANES suggested that OBS was strongly negatively associated with gallstone, stressing the importance of dietary and lifestyle factors in daily life.

## Data Availability

Publicly available datasets were analyzed in this study. This data can be found here: https://www.cdc.gov/nchs/nhanes.

## References

[B1] Ahmadi N. Eshaghian S. Huizenga R. Sosnin K. Ebrahimi R. Siegel R. (2011). Effects of intense exercise and moderate caloric restriction on cardiovascular risk factors and inflammation. Am. J. Med. 124 (10), 978–982. 10.1016/j.amjmed.2011.02.032 21798505

[B2] Aune D. Vatten L. J. (2016). Diabetes mellitus and the risk of gallbladder disease: a systematic review and meta-analysis of prospective studies. J. Diabetes. Complications. 30(2):368–373. 10.1016/j.jdiacomp.2015.11.012 26684168

[B3] Bellows C. F. Berger D. H. Crass R. A. (2005). Management of gallstones. Am. Fam. Physician 72 (4), 637–642. 16127953

[B4] Belotto M. F. Magdalon J. Rodrigues H. G. Vinolo M. A. R. Curi R. Pithon-Curi T. C. (2010). Moderate exercise improves leucocyte function and decreases inflammation in diabetes. Clin. Exp. Immunol. 162 (2), 237–243. 10.1111/j.1365-2249.2010.04240.x 20846161 PMC2996590

[B5] Biddinger S. B. Haas J. T. Yu B. B. Bezy O. Jing E. Zhang W. (2008). Hepatic insulin resistance directly promotes formation of cholesterol gallstones. Nat. Med. 14 (7), 778–782. 10.1038/nm1785 18587407 PMC2753607

[B6] Bjork L. Jenkins N. T. Witkowski S. Hagberg J. M. (2012). Nitro-oxidative stress biomarkers in active and inactive men. Int. J. Sports Med. 33 (4), 279–284. 10.1055/s-0032-1301891 22377943

[B7] Carey M. C. Small D. M. (1978). The physical chemistry of cholesterol solubility in bile. Relationship to gallstone formation and dissolution in man. J. Clin. Invest. 61 (4), 998–1026. 10.1172/JCI109025 659586 PMC372618

[B8] Cheng Y.-T. Yang C.-C. Shyur L.-F. (2016). Phytomedicine-Modulating oxidative stress and the tumor microenvironment for cancer therapy. Pharmacol. Res. 114, 128–143. 10.1016/j.phrs.2016.10.022 27794498

[B9] Darley-Usmar V. Halliwell B. (1996). Blood radicals: reactive nitrogen species, reactive oxygen species, transition metal ions, and the vascular system. Pharm. Res. 13 (5), 649–662. 10.1023/a:1016079012214 8860419

[B10] Fang Y.-Z. Yang S. Wu G. (2002). Free radicals, antioxidants, and nutrition. Nutrition 18 (10), 872–879. 10.1016/s0899-9007(02)00916-4 12361782

[B11] Floyd R. A. (1990). Role of oxygen free radicals in carcinogenesis and brain ischemia. FASEB J. 4 (9), 2587–2597. 10.1096/fasebj.4.9.2189775 2189775

[B12] Genova M. L. Lenaz G. (2015). The interplay between respiratory supercomplexes and ROS in aging. Antioxid. Redox Signal 23 (3), 208–238. 10.1089/ars.2014.6214 25711676

[B13] Hernández-Ruiz Á. García-Villanova B. Guerra-Hernández E. Amiano P. Ruiz-Canela M. Molina-Montes E. (2019). A review of *a priori* defined oxidative balance scores relative to their components and impact on health outcomes. Nutrients 11 (4), 774. 10.3390/nu11040774 30987200 PMC6520884

[B14] Hernández-Ruiz Á. García-Villanova B. Guerra-Hernández E. J. Carrión-García C. J. Amiano P. Sánchez M.-J. (2022). Oxidative balance scores (OBSs) integrating nutrient, food and lifestyle dimensions: development of the NutrientL-OBS and FoodL-OBS. Antioxidants (Basel) 11 (2), 300. 10.3390/antiox11020300 35204183 PMC8868253

[B15] Hou W. Han T. Sun X. Chen Y. Xu J. Wang Y. (2022). Relationship between carbohydrate intake (quantity, quality, and time eaten) and mortality (total, cardiovascular, and diabetes): assessment of 2003-2014 national health and nutrition examination survey participants. Diabetes Care 45 (12), 3024–3031. 10.2337/dc22-0462 36174119

[B16] Hu J. Zou H. Qiao X. Wang Y. Lv M. Zhang K. (2024). The relationship between oxidative balance scores and chronic diarrhea and constipation: a population-based study. BMC Public Health 24 (1), 1366. 10.1186/s12889-024-18683-8 38773415 PMC11106991

[B17] Iyama T. Takasuga A. Azuma M. (1996). beta-Carotene accumulation in mouse tissues and a protective role against lipid peroxidation. Int. J. Vitam. Nutr. Res. 66 (4), 301–305. 8979157

[B18] Kaur T. Kaur S. (2010). Pathophysiological conditions in cholelithiasis formation in North Indian population: spectroscopic, biophysical, and biochemical study. Biol. Trace Elem. Res. 138 (1-3), 79–89. 10.1007/s12011-010-8618-0 20186501

[B19] Ke R. He Y. Chen C. (2023). Association between oxidative balance score and kidney stone in United States adults: analysis from NHANES 2007-2018. Front. Physiol. 14, 1275750. 10.3389/fphys.2023.1275750 38028789 PMC10654971

[B20] Kim S. Y. (2022). Oxidative stress and gender disparity in cancer. Free Radic. Res. 56 (1), 90–105. 10.1080/10715762.2022.2038789 35118928

[B21] Koppisetti S. Jenigiri B. Terron M. P. Tengattini S. Tamura H. Flores L. J. (2008). Reactive oxygen species and the hypomotility of the gall bladder as targets for the treatment of gallstones with melatonin: a review. Dig. Dis. Sci. 53 (10), 2592–2603. 10.1007/s10620-007-0195-5 18338264

[B22] Lammert F. Gurusamy K. Ko C. W. Miquel J.-F. Méndez-Sánchez N. Portincasa P. (2016). Gallstones. Nat. Rev. Dis. Prim. 2, 16024. 10.1038/nrdp.2016.24 27121416

[B23] Le Lay S. Simard G. Martinez M. C. Andriantsitohaina R. (2014). Oxidative stress and metabolic pathologies: from an adipocentric point of view. Oxid. Med. Cell. Longev. 2014, 908539. 10.1155/2014/908539 25143800 PMC4131099

[B24] Liu X. Liu X. Wang Y. Zeng B. Zhu B. Dai F. (2023). Association between depression and oxidative balance score: national health and nutrition examination survey (NHANES) 2005-2018. J. Affect Disord. 337, 57–65. 10.1016/j.jad.2023.05.071 37244542

[B25] Liu X.-J. Yang W. Qi J.-S. (2012). Oxidative stress and Alzheimer's disease. Sheng Li Xue Bao 64 (1), 87–95. 22348966

[B26] Liu X.-T. Hu J. (2002). Relationship between bilirubin free radical and formation of pigment gallstone. World J. Gastroenterol. 8 (3), 413–417. 10.3748/wjg.v8.i3.413 12046060 PMC4656411

[B27] López-Lluch G. Santos-Ocaña C. Sánchez-Alcázar J. A. Fernández-Ayala D. J. M. Asencio-Salcedo C. Rodríguez-Aguilera J. C. (2015). Mitochondrial responsibility in ageing process: innocent, suspect or guilty. Biogerontology 16 (5), 599–620. 10.1007/s10522-015-9585-9 26105157

[B28] Marcus S. N. Heaton K. W. (1986). Effects of a new, concentrated wheat fibre preparation on intestinal transit, deoxycholic acid metabolism and the composition of bile. Gut 27 (8), 893–900. 10.1136/gut.27.8.893 3015748 PMC1433372

[B29] Marlatt M. W. Lucassen P. J. Perry G. Smith M. A. Zhu X. (2008). Alzheimer's disease: cerebrovascular dysfunction, oxidative stress, and advanced clinical therapies. J. Alzheimers Dis. 15 (2), 199–210. 10.3233/jad-2008-15206 18953109 PMC2774209

[B30] Marschall H. U. Einarsson C. (2007). Gallstone disease. J. Intern Med. 261 (6), 529–542. 10.1111/j.1365-2796.2007.01783.x 17547709

[B31] McMichael A. J. Baghurst P. A. Scragg R. K. (1992). A case-control study of smoking and gallbladder disease: importance of examining time relations. Epidemiology 3 (6), 519–522. 10.1097/00001648-199211000-00010 1420518

[B32] Poljsak B. Šuput D. Milisav I. (2013). Achieving the balance between ROS and antioxidants: when to use the synthetic antioxidants. Oxid. Med. Cell. Longev. 2013, 956792. 10.1155/2013/956792 23738047 PMC3657405

[B33] Portincasa P. Moschetta A. Palasciano G. (2006). Cholesterol gallstone disease. Lancet 368 (9531), 230–239. 10.1016/S0140-6736(06)69044-2 16844493

[B34] Qu H. (2023). The association between oxidative balance score and periodontitis in adults: a population-based study. Front. Nutr. 10, 1138488. 10.3389/fnut.2023.1138488 37187879 PMC10178495

[B35] Reuter S. Gupta S. C. Chaturvedi M. M. Aggarwal B. B. (2010). Oxidative stress, inflammation, and cancer: how are they linked? Free Radic. Biol. Med. 49 (11), 1603–1616. 10.1016/j.freeradbiomed.2010.09.006 20840865 PMC2990475

[B36] Rock E. Astier C. Lab C. Vignon X. Gueux E. Motta C. (1995). Dietary magnesium deficiency in rats enhances free radical production in skeletal muscle. J. Nutr. 125 (5), 1205–1210. 10.1093/jn/125.5.1205 7738680

[B37] Sanikidze T. Chikvaidze E. (2016). Role of the free radicals in mechanisms of gallstone formation: an EPR study. Radiat. Prot. Dosim. 172 (1-3), 317–324. 10.1093/rpd/ncw237 27574326

[B38] Schwesinger W. H. Kurtin W. E. Page C. P. Stewart R. M. Johnson R. (1999). Soluble dietary fiber protects against cholesterol gallstone formation. Am. J. Surg. 177 (4), 307–310. 10.1016/s0002-9610(99)00047-1 10326849

[B39] Senoner T. Dichtl W. (2019). Oxidative stress in cardiovascular diseases: still a therapeutic target? Nutrients 11 (9), 2090. 10.3390/nu11092090 31487802 PMC6769522

[B40] Shabanzadeh D. M. (2018). Incidence of gallstone disease and complications. Curr. Opin. Gastroenterol. 34 (2), 81–89. 10.1097/MOG.0000000000000418 29256915

[B41] Shebl F. M. Andreotti G. Rashid A. Gao Y. T. Yu K. Shen M. C. (2010). Diabetes in relation to biliary tract cancer and stones: a population-based study in Shanghai, China. Br. J. Cancer 103 (1), 115–119. 10.1038/sj.bjc.6605706 20517308 PMC2905288

[B42] Shiesh S. C. Chen C. Y. Lin X. Z. Liu Z. A. Tsao H. C. (2000). Melatonin prevents pigment gallstone formation induced by bile duct ligation in Guinea pigs. Hepatology 32 (3), 455–460. 10.1053/jhep.2000.16332 10960434

[B43] Singh N. Kazim S. N. Sultana R. Tiwari D. Borkotoky R. Kakati S. (2019). Oxidative stress and deregulations in base excision repair pathway as contributors to gallbladder anomalies and carcinoma - a study involving North-East Indian population. Free Radic. Res. 53 (5), 473–485. 10.1080/10715762.2019.1606423 31117842

[B44] Stafford R. E. Mak I. T. Kramer J. H. Weglicki W. B. (1993). Protein oxidation in magnesium deficient rat brains and kidneys. Biochem. Biophys. Res. Commun. 196 (2), 596–600. 10.1006/bbrc.1993.2291 8240333

[B45] Strehlow K. Rotter S. Wassmann S. Adam O. Grohé C. Laufs K. (2003). Modulation of antioxidant enzyme expression and function by estrogen. Circ. Res. 93 (2), 170–177. 10.1161/01.RES.0000082334.17947.11 12816884

[B46] Tian X. Xue B. Wang B. Lei R. Shan X. Niu J. (2022). Physical activity reduces the role of blood cadmium on depression: a cross-sectional analysis with NHANES data. Environ. Pollut. 304, 119211. 10.1016/j.envpol.2022.119211 35341822

[B47] Topinka J. Binkova B. Sram R. J. Erin A. N. (1989). The influence of alpha-tocopherol and pyritinol on oxidative DNA damage and lipid peroxidation in human lymphocytes. Mutat. Res. 225 (3), 131–136. 10.1016/0165-7992(89)90130-9 2927430

[B48] Weiss S. J. (1989). Tissue destruction by neutrophils. N. Engl. J. Med. 320 (6), 365–376. 10.1056/NEJM198902093200606 2536474

[B49] Willi C. Bodenmann P. Ghali W. A. Faris P. D. Cornuz J. (2007). Active smoking and the risk of type 2 diabetes: a systematic review and meta-analysis. JAMA 298 (22), 2654–2664. 10.1001/jama.298.22.2654 18073361

[B50] Wu C. Ren C. Song Y. Gao H. Pang X. Zhang L. (2023). Gender-specific effects of oxidative balance score on the prevalence of diabetes in the US population from NHANES. Front. Endocrinol. (Lausanne) 14, 1148417. 10.3389/fendo.2023.1148417 37214249 PMC10194026

[B51] Wu G. Meininger C. J. (2002). Regulation of nitric oxide synthesis by dietary factors. Annu. Rev. Nutr. 22, 61–86. 10.1146/annurev.nutr.22.110901.145329 12055338

[B52] Xiong T. Chen Z. Yi J. Yu T. Wang K. (2025). Higher levels of oxidative balance score linked to lower risk of gallstones: findings from the 2017-2020 National Health and Nutrition Examination Survey. Front. Nutr. 12, 1521882. 10.3389/fnut.2025.1521882 39944962 PMC11816672

[B53] Xu W. Mu D. Wang Y. Wang Y. Wang C. Zhang X. (2024). Association between oxidative balance score and sarcopenia in US adults: NHANES 2011-2018. Front. Nutr. 11, 1342113. 10.3389/fnut.2024.1342113 38721026 PMC11076835

[B54] Yang G. Q. Wang S. Z. Zhou R. H. Sun S. Z. (1983). Endemic selenium intoxication of humans in China. Am. J. Clin. Nutr. 37 (5), 872–881. 10.1093/ajcn/37.5.872 6846228

[B55] Zhang P. Li T. Wu X. Nice E. C. Huang C. Zhang Y. (2020). Oxidative stress and diabetes: antioxidative strategies. Front. Med. 14 (5), 583–600. 10.1007/s11684-019-0729-1 32248333

[B56] Zhang W. Peng S.-F. Chen L. Chen H.-M. Cheng X.-E. Tang Y.-H. (2022). Association between the oxidative balance score and telomere length from the national health and nutrition examination survey 1999-2002. Oxid. Med. Cell. Longev. 2022, 1345071. 10.1155/2022/1345071 35186180 PMC8850082

[B57] Zhu H. Jin L. Zhang Z. Lu C. Jiang Q. Mou Y. (2025). Oxidative balance scores and gallstone disease: mediating effects of oxidative stress. Nutr. J. 24 (1), 4. 10.1186/s12937-025-01073-0 39789597 PMC11720334

