# Supplementary material for: Association between oxidative balance score and gallstone in US adults: a cross-sectional study
Source: Front Aging. 2025 Jul 15;6:1621107. doi: 10.3389/fragi.2025.1621107 (PMC12303946; doi:10.3389/fragi.2025.1621107)
Supplement: Supplementary file 1 [file Table1.docx]

Supplementary Material

Association between oxidative balance score and gallstone in US adults:

a cross-sectional study

Xiaoya Chen^1,2, †^, Xiongwei Huo^2,†^, Changchun Ye^2^, Zhengshui Xu^1^, Zilu Chen^2,^*, Shiyuan Liu ^1*^

^1^Department of Thoracic Surgery, The Second Affiliated Hospital of Xi'an Jiaotong University, Xi'an, Shaanxi, 710004, China

^2^Department of General Surgery, The First Affiliated Hospital of Xi'an Jiaotong University, Xi'an, Shaanxi, 710061, China

*** Correspondence:**Corresponding Author
Zilu Chen (lucychenzz@stu.xjtu.edu.cn)

Shiyuan Liu ([liushiyuan@xjtu.edu.cn](mailto:liushiyuan@xjtu.edu.cn))

† These authors contributed equally to this work

TABLE S1 Oxidative balance score calculation

| OBS components | Property | Male | | | Female | | |
| --- | --- | --- | --- | --- | --- | --- | --- |
|  |  | 0 | 1 | 2 | 0 | 1 | 2 |
| Dietary OBS components | | | | | | | |
| Dietary fiber(g/d) | A | <11.9 | 11.9-19.7 | ≥19.7 | <10.1 | 10.1-17.1 | ≥17.1 |
| Carotene (mcg/d) | A | <392 | 392-1507.7 | ≥1507.7 | <407.67 | 407.67-1831 | ≥1831 |
| Niacin(mg/d) | A | <20.8 | 20.8-31.3 | ≥31.3 | <14.90 | 14.90-22.65 | ≥22.65 |
| Riboflavin(mg/d) | A | <1.57 | 1.57-2.36 | ≥2.36 | <1.23 | 1.23-1.81 | ≥1.81 |
| Total folate(mcg/d) | A | <281.3 | 281.3-443 | ≥443 | <227 | 227-348 | ≥348 |
| Vitamin B6(mg/d) | A | <1.53 | 1.53-2.36 | ≥2.36 | <1.13 | 1.13-1.80 | ≥1.80 |
| Vitamin B12(mcg/d) | A | <2.82 | 2.82-5.49 | ≥5.49 | <1.93 | 1.93-3.76 | ≥3.76 |
| Total fat (g/d) | P | ≥111.18 | 71.31-111.18 | <71.31 | ≥84.95 | 55.64-84.95 | <55.64 |
| Vitamin C(mg/d) | A | <27.93 | 27.93-86.27 | ≥86.27 | <25.7 | 25.7-80.37 | ≥80.37 |
| Vitamin E(mg/d) | A | <6.44 | 6.44-10.63 | ≥10.63 | <5.46 | 5.46-8.97 | ≥8.97 |
| Selenium(mcg/d) | A | <95.3 | 95.3-142.63 | ≥142.63 | <69.1 | 69.1-104.5 | ≥104.5 |
| Iron (mg/d) | P | ≥16.45 | 11.11-16.45 | <11.11 | ≥12.71 | 8.31-12.71 | <8.31 |
| Magnesium(mg/d) | A | <246 | 246-361 | ≥361 | <200.0 | 200.0-292.33 | ≥292.33 |
| Calcium(mg/d) | A | <687.33 | 687.33-1108.67 | ≥1108.67 | <568.67 | 568.67-912 | ≥912 |
| Copper(mg/d) | A | <0.92 | 0.92-1.37 | ≥1.37 | <0.76 | 0.76-1.15 | ≥1.15 |
| Zinc(mg/d) | A | <8.53 | 8.53-13.18 | ≥13.18 | <6.17 | 6.17-9.57 | ≥9.57 |
| Lifestyle OBS components | | | | | | | |
| Physical activity  (MET-minute/week) | A | <1,320 | 1,320-4,800 | ≥4,880 | <840 | 840-2,880 | ≥2,880 |
| Alcohol(g/d) | P | ≥30 | 0-30 | None | ≥15 | 0-15 | None |
| Body mass index(kg/m²) | P | ≥31.1 | 26.2-31.1 | <26.2 | <26 | 26-32.2 | ≥32.2 |
| Cotinine(ng/mL) | P | ≥0.799 | 0.016-0.799 | <0.016 | ≥0.079 | 0.011-0.079 | <0.011 |

A stood for the antioxidant, P for the pro-oxidant, MET for the metabolic equivalent.

Table S2 The definition of the other comorbidities.

| Diseases | Definition |
| --- | --- |
| Diabetes | 1.Medical diagnosis of diabetes as recorded by the patient’s healthcare provider or current use of insulin or diabetic pills. |
|  | 2.glycohemoglobin A1c (HbA1c) level higher than 6.5%. |
|  | 3.fasting blood glucose level equal to or higher than 7.0 mmol/L. |
| Hypertension | self-reported diagnosis of hypertension. |
| Asthma | self-reported diagnosis of asthma. |
| Coronary heart disease (CHD) | self-reported diagnosis of coronary heart disease. |

Table S3  Additional baseline characteristics based on the presence or absence of gallstone.

| Characteristic | Overall N=4,376(100%) | Normal N=3,948 (89.71%) | Gallstone N=428(10.29%) | *p* value |
| --- | --- | --- | --- | --- |
| Age(years), Mean±SE | 47.34±0.67 | 46.32 ± 0.72 | 56.19 ± 1.00 | **<0.001** |
| Gender, n(%) |  |  |  | **<0.001** |
| Male | 2,201(50.22) | 2,075 (52.83) | 126 (27.50) |  |
| Female | 2,175(49.78) | 1,873 (47.17) | 302 (72.50) |  |
| Race, n(%) |  |  |  | **0.021** |
| Mexican American | 500(7.73) | 450 (7.88) | 50 (6.39) |  |
| Non-Hispanic White | 1,698(67.62) | 1,497 (66.83) | 201 (74.46) |  |
| Non-Hispanic Black | 1,045(9.39) | 972 (9.82) | 73 (5.65) |  |
| Other | 1,133(15.26) | 1,029 (15.47) | 104 (13.50) |  |
| Education level, n(%) |  |  |  | 0.218 |
| Below high school | 628(7.87) | 572 (8.01) | 56 (6.70) |  |
| High school | 1,011(25.83) | 903 (25.26) | 108 (30.79) |  |
| High school above | 2,737(66.30) | 2,473 (66.73) | 264 (62.51) |  |
| PIR group, n(%) |  |  |  | 0.050 |
| <=1.3 | 1,129(16.34) | 1,029 (16.45) | 100 (15.36) |  |
| 1.3-3.5 | 1,646(33.22) | 1,476 (32.44) | 170 (40.00) |  |
| >3.5 | 1,601(50.44) | 1,443 (51.11) | 158 (44.65) |  |
| Marital status, n(%) |  |  |  | **<0.001** |
| Married/Living with Partner | 2,630(64.34) | 2,369 (64.36) | 261 (64.18) |  |
| Widowed/Divorced/Separated | 891(16.53) | 778 (15.79) | 113 (23.01) |  |
| Never married | 855(19.12) | 801 (19.85) | 54 (12.81) |  |
| Energy intake(kcal), Mean±SE | 2,107.23±12.92 | 2,136.60 ± 12.18 | 1,851.11 ± 59.39 | **<0.001** |
| Cholesterol(mmol/L),Mean±SE | 4.86±0.04 | 4.85 ± 0.04 | 4.94 ± 0.07 | 0.207 |
| Cotinine(ng/mL),Mean±SE | 50.14±5.00 | 50.25 ± 5.35 | 49.23 ± 6.94 | 0.898 |
| Alcohol(g/d),Mean±SE | 1.99±0.07 | 2.06 ± 0.08 | 1.42 ± 0.11 | **<0.001** |
| MET(minute/week),Mean±SE | 4,175.42±172.66 | 4,200.39±179.01 | 3,957.71 ± 507.33 | 0.648 |
| BMI(kg/m²), Mean±SE | 29.46±0.19 | 29.12 ± 0.18 | 32.43 ± 0.54 | **<0.001** |
| Asthma, n(%) |  |  |  | 0.190 |
| Yes | 684(14.13) | 599 (13.84) | 85 (16.66) |  |
| No | 3,692(85.87) | 3,349 (86.16) | 343 (83.34) |  |
| CHD, n(%) |  |  |  | **0.021** |
| Yes | 176(3.86) | 140 (3.63) | 36 (5.87) |  |
| No | 4,200(96.14) | 3,808 (96.37) | 392 (94.13) |  |
| Hypertension, n(%) |  |  |  | **<0.001** |
| Yes | 1,534(29.66) | 1,322 (28.23) | 212 (42.16) |  |
| No | 2,842(70.34) | 2,626 (71.77) | 216 (57.84) |  |
| Diabetes, n(%) |  |  |  | **<0.001** |
| Yes | 785(13.87) | 666 (12.64) | 119 (24.63) |  |
| No | 3,591(86.13) | 3,282 (87.36) | 309 (75.37) |  |
| Total OBS | 20.83±0.18 | 20.96±0.17 | 19.75±0.62 | **<0.001** |

For continuous variables: survey-weighted mean (SE), P-value was by survey-weighted linear regression  
For categorical variables: survey-weighted percentage (95% CI) , P-value was by survey-weighted Chi-square test

PIR, income to poverty ratio; CHD, coronary heart disease; BMI, body mass index; MET, metabolic equivalent; OBS, oxidative balance score.

Table S4: Sensitivity analyses to evaluate the effects of individual OBS components on the gallstone.

| OBS | Gallstone | |  |
| --- | --- | --- | --- |
| OBS adjusted for model 3 | OR | 95% CI | p-value |
| 1OBS excluding physical activity | 0.96 | 0.94, 0.98 | <0.001 |
| 2OBS excluding alcohol | 0.96 | 0.94, 0.98 | <0.001 |
| 3OBS excluding body mass index | 0.97 | 0.95, 0.99 | 0.010 |
| 4OBS excluding cotinine | 0.97 | 0.95, 0.99 | <0.001 |
| 5OBS excluding dietary fiber | 0.96 | 0.94, 0.98 | <0.001 |
| 6OBS excluding Carotene | 0.96 | 0.94, 0.98 | <0.001 |
| 7OBS excluding Riboflavin | 0.96 | 0.94, 0.98 | <0.001 |
| 8OBS excluding Niacin | 0.96 | 0.94, 0.98 | <0.001 |
| 9OBS excluding vitamin b6 | 0.96 | 0.94, 0.98 | <0.001 |
| 10OBS excluding total folate | 0.96 | 0.94, 0.98 | <0.001 |
| 11OBS excluding vitamin B12 | 0.96 | 0.94, 0.98 | <0.001 |
| 12OBS excluding vitamin C | 0.96 | 0.94, 0.98 | <0.001 |
| 13OBS excluding vitamin E | 0.96 | 0.94, 0.98 | <0.001 |
| 14OBS excluding Calcium | 0.96 | 0.94, 0.98 | <0.001 |
| 15OBS excluding Magnesium | 0.96 | 0.94, 0.98 | <0.001 |
| 16OBS excluding Zinc | 0.96 | 0.94, 0.98 | <0.001 |
| 17OBS excluding Copper | 0.96 | 0.94, 0.98 | <0.001 |
| 18OBS excluding Selenium | 0.96 | 0.94, 0.98 | <0.001 |
| 19OBS excluding total fat | 0.96 | 0.94, 0.98 | <0.001 |
| 20OBS excluding Iron | 0.97 | 0.95, 0.98 | <0.001 |
